# Double Burden of COVID-19 Pandemic and Military Occupation: Mental Health Among a Palestinian University Community in the West Bank

**DOI:** 10.5334/aogh.3007

**Published:** 2020-10-08

**Authors:** Rula Ghandour, Rasha Ghanayem, Farah Alkhanafsa, Ayah Alsharif, Hiba Asfour, Aisha Hoshiya, Amani Masalmeh, Muna Nadi, Laila Othman, Sameera Ryahe, Yasmeen Wahdan, Shatha Wahsh, Ala’a Yamani, Rita Giacaman

**Affiliations:** 1 Birzeit University, PS

## Abstract

**Background:**

The Covid-19 pandemic created major global health crises, with serious effects on all aspects of life. The pandemic reached the Israeli occupied West Bank of Palestine in early March 2020, and lockdown immediately ensued.

**Objectives:**

To assess the prevalence and predictors of distress and insecurity among Birzeit University’s community during the COVID-19 pandemic and lockdown.

**Methods:**

An online survey completed in March-April 2020 using standardized and previously validated distress and insecurity scales. The survey was placed on the University portal accessed by students, faculty and employees, and was sent by email to faculty and employees. Data were weighted to reflect the University community’s distribution.

**Findings:**

There were 1,851 participants in the study: 84% were undergraduate students, 10% graduate students, and 6% faculty and employees. Sixty two percent were women. Ages ranged from 17 to 70 years (mean 24 ± 9.7). Prevalence of moderate/high distress and insecurity were 40% and 48% respectively. Multiple logistic regression revealed that women, those under 35 years old and those with worse reported income, had significantly higher odds of distress and insecurity compared to their counterparts. Undergraduate students or living with a person at home with high risk of illness with COVID-19 were associated with higher odds of distress compared to their counterparts (OR = 1.56, 95%CI[1.13–2.15]) and (OR = 1.34, 95%CI[1.11–1.62]) respectively. A COVID-19 worry score was significantly associated with higher odds of distress and insecurity (OR = 1.77, 95%CI[1.46–2.14]) and (OR = 4.3, 95%CI[3.53–5.23]) respectively.

**Conclusion:**

This study emphasizes the need to pay attention not only to physical health but also to mental health during the COVID-19 pandemic, especially among young people, women, those with lower economic status, and those living with high risk persons during the pandemic. We hope that this study will inform the policies and interventions of the Palestinian Authority, local non-governmental organization, international groups working in the occupied Palestinian territory, and beyond.

## Introduction

The Covid-19 pandemic has reached most of the globe, creating major health crises [1]. The global scale of continuing transmission, and high numbers of deaths, infection, and mortality, are major causes of concern [2], with the pandemic declared a worldwide public health emergency [3]. By September 3^rd^, 2020, the World Health Organization had identified 25,884,895 confirmed cases globally, with 859,130 deaths from infection [4]. To address this pandemic and contain it, widespread lockdown in many countries ensued [5], with serious effects on all aspects of life [6].

Attention was initially placed on the effects of the pandemic on older people as the risk of dying from the infection increases with age [7], with adverse outcomes linked to males, smoking and cardio-metabolic comorbidity [8]. However, the pandemic exposed racial and other disparities, with the risk of disease and death exacerbated by inequalities [9,10].

The pandemic, compounded by lockdowns, created the conditions for fears and worries, distress and anxieties to rise among all [11], with effects on different age groups and genders on mental health [12,13], including on front line workers, young children, and college students [14]. A study of undergraduate students reported that isolation from social networks and the lack of interaction with others were associated with negative mental health [15]. At the peak of the pandemic in China, the prevalence of psychological distress in the general population was as high as 35% [16]. A systematic review, which included 43 studies [17], revealed worsening psychiatric symptoms among those with pre-existing psychiatric disorders. Studies of health care workers found increased depressive symptoms, anxiety, psychological distress, and poor sleep quality. Studies of the general public revealed lower psychological well-being and higher scores of anxiety and depression compared to before the COVID-19 pandemic.

When the pandemic reached the Israeli occupied Palestinian West Bank on March 5^th^ 2020, the mental health status of Palestinians was already compromised because of the political context [18]. Since 1967, and the fall of the West Bank (including East Jerusalem) and the Gaza Strip, under Israeli military rule, Palestinians have endured chronic exposure to political violence, oppression, subjugation, and lack of freedom [19]. Generations of Palestinians have suffered human rights violations, including land confiscation, displacement, control by the Israeli army of the movement of people and goods from one area to another – and outside the country – death, injury, disability, imprisonment, the lack of freedom, and injustice. The Palestinian Authority (PA) has little authority in practice, is burdened by starvation for funds and dependence on foreign aid [20], and was unable to fulfill the basic needs of the population even before the pandemic reached the West Bank.

It is in this context that Palestinian West Bankers experienced the pandemic in early March 2020, adding insult to injury. The PA immediately implemented lockdown [21], with all staying at home, except for buying foods or medicine or in case of emergency. It seemed like the pandemic was under control. However, by the beginning of April, the number of infected cases began to rise, as Palestinian workers in Israel returned home during the Passover holidays, many infected [22], and with Israel becoming the largest source for the virus spread in the West Bank at the time [23]. By May, the PA extended lockdown [24] which was strictly implemented given years of Israeli military occupation which stunted Palestinian health services [20], and the limited powers the PA has in accessing all areas of the West Bank due to the complete control of 60% of West Bank land by Israel [19].

The severity of the measures to curb the spread of the virus have had serious effects on many families’ already fragile financial situation, and exacerbated mental health problems [18]. Reports indicate a worsening of the job market because of lockdown, and a rise in unemployment rates [23]. Thus the lockdown began to be protested by the end of May 2020, having disrupted already fragile local economies, with many households falling below the poverty line. This led to substantial relaxation of lockdown conditions [15]. It is in this context of the Palestinian double captivity (Israeli military occupation and the COVID-19 pandemic and lockdown) that this research on the effects of the pandemic on the mental health of Palestinians living in the West Bank was completed. The aim of this study was to investigate the mental health status of the Birzeit University community during the COVID-19 pandemic and lockdown using locally developed and validated distress and insecurity measures.

## Methods

This is a cross-sectional exploratory study using an online survey tool completed in late March to early April 2020. Participants included undergraduate and post graduate students, faculty, and employees. The survey tool was built using KOBO Toolbox® software. The survey link was placed on the main student/employee/faculty platform (Ritaj), and excluded guards and cleaners as they do not have access to either the Ritaj University platform or the University’s email system. In addition, a link was sent to all faculty and employees by email once.

### Tools and Instruments

The study tool was based on standardized validated distress and insecurity scales previously developed and tested by the Institute of Community and Public Health at Birzeit University (ICPH/BZU) [25,26,27]. ICPH/BZU faculty and researchers have and continue to develop measures to assess mental health in wars and conflicts which are compatible with the way in which we conceptualize health and disease in chronic warlike conditions. Using a bio-psychosocial approach, we define health and disease as a continuum between Ease and Dis-ease, as opposed to the dichotomous conceptualization of either healthy or sick, which pathologizes the suffering people endure in political conflicts. We include a domain related to suffering between health and disease. This domain includes items such as human insecurity, deprivation, humiliation, and uncertainty etc., the traumas of war, which affect mental health and wellbeing negatively, but do not necessarily lead to diagnosable psychiatric conditions. We call such suffering the ‘invisible wounds inside’ which over the life course, can lead to disease. From a public health perspective, research and interventions need to focus on the in between conditions, to prevent people from moving towards the Dis-ease end of the continuum [28]. The other rationale for developing local scales is our experience indicating that distress is manifested differently in different contexts and cultures. Our experience also indicates that sometimes international scales are less sensitive to changes in health and mental health status over time or as a result of exposure to particularly violent incidents compared to our locally developed distress and insecurity scales [26].

Given this framework, distress is defined as a mental health condition characterized by emotional suffering, frustration, anxiousness, sadness, anger, and incapacitation and related feelings. Insecurity contains psychological and social components, focusing on the sense of home, link to community, and positive and hopeful sense of the future (see Supplement for the items found in the scales). The distress scale contains 12 items and the insecurity scale contains 10 items, with likert scale responses ranging from 0 (not at all) to 3 (all the time). Both scales had very good internal consistency (Cronbach’s alpha: 0.90 and 0.87 for distress and insecurity respectively). For this survey, exploratory factor analysis was also conducted on these scales with varimax rotation, with both scale items maintaining their validity and factor structure. Furthermore, a COVID-19 worry scale was created based on three questions: are you worried about yourself and your family because of the COVID-19 pandemic (1) socially, (2) economically, or (3) health wise, and with worry defined as feelings and disturbing thoughts about something bad which may be happening, or concern over experiencing possible troubles. The scale had good internal consistency with a Cronbach’s alpha of 0.77. Each scale item had a value between 0 (not at all) and 3 (all the time). The score of all items was summed and converted to 100. The mean COVID-19 worry score was used as a cut-off point creating a binary variable. Both continuous and categorical worry score variables were entered into the regression model, first as a continuous, then as a categorical variable, with the same results obtained.

Information on age, gender, status (student, employee or administrative staff, faculty, or academics), and perceived income compared to others, district of domicile, and locality (urban, rural, and Palestinian refugee camp) were collected. In addition, information was also collected on living with a person at high risk of illness with COVID-19, such as the elderly, persons with non-communicable disease, or those who are immuno-compromised.

### Distress and insecurity scale calculation

Some scale items had responses of “I do not know”, and were treated as missing. A weighted average score was calculated for each scale based on the valid responses (i.e the scores were calculated for each participant for each scale based on valid responses to the scale items). Missing values within scale items were minimal (98% answered 10–12 items of the distress scale and more than 95% of respondents answered 8–10 items of the insecurity scale). Final scores for distress and insecurity were calculated (Supplement). The score ranged between 0 and 100 using a cutoff point of 50, each scale was divided into two categories (low level and moderate to high levels).

### Weighting

As this is an online survey making randomization difficult, the final sample was corrected based on the general Birzeit University community distribution. Before weighting, 64% (N = 1,178) of the respondents were women and 34% (N = 673) were men; 87% (N = 1441) were undergraduate, and 10% (N = 195) graduate students, while 11% (N = 215) were faculty and employees. Data were weighted based on gender and status distributions. Relative weights were calculated for each of the three groups (undergraduate students, postgraduate students, and faculty and employees), and by gender. First, the probability of being sampled among each status category and gender was calculated, then the base weight was calculated as the inverse of the sampling probability; then the relative weights were calculated by dividing each base weight by the average base weights. Relative weights were applied to the dataset and weighted analysis was conducted using SPSS 26®. As can be seen under results, the weighted sample did not differ substantially from the unweighted.

### Data manipulation

Age was recoded using a cutoff point of 35 years with a new age variable composed of two values (< 35 years and 35+). We hypothesized that participants 35 years old or less had not experienced lockdown previously and may be more negatively affected by lockdown, compared those more than 35 years old who were around 15 years old or more during the Second Palestinian Uprising, and who have experienced curfews and lockdowns. District was recoded into North, Center, and South West Bank, in addition to the category ‘other’ for those attending Birzeit University with permanent residences outside the West Bank, for example, in Jordan, Gulf countries, or Palestinians with Israeli citizenship living in Israel. For the regression analysis, the status of participants was combined into two categories: faculty, employees, and postgraduate students together based on the assumption that they share common characteristics including independence from parents, being married and having work and families, as is the case with many post-graduate students at the University; and undergraduate students in another category, who generally are unmarried, do not work and live with and depend on parents.

### Statistical analysis

Bivariate analysis was conducted between distress and insecurity as dependent variables followed by two multiple logistic regressions with the distress and insecurity as dependent variables in each model and significant predictors from the bivariate analysis. Those included age, gender, status, perceived income, living district, living with a high-risk person at home and being worried from the COVID-19 pandemic.

### Ethics

This research was approved by the Research Ethics Committee (REC) at the Institute of Community and Public Health, Birzeit University (Ethical Approval Reference number: 2020[1]). Informed consent was obtained electronically. All authors agreed on the order of the authorship of this paper.

## Results

A total of 1,851 questionnaires were collected, with a response rate of 97% among those who accessed the link and read the consent form. The overall response rate was 12%, with 11% for undergraduate students, 12% for graduate students, and 24% for faculty and employees. Weighted analysis revealed that the sample contained 62% women and 38% men, 84% undergraduate students, 10% postgraduate students, and 6% faculty and employees (Table 1). Mean age was 24 ± 9.7 years old, with 94% under the age of 35 years old. The majority were living in the central West Bank (78%), 11% in the north, 7% in the south West Bank, and 4% reported living outside the West Bank. Fifty one percent were from urban areas with few living in Palestinian refugee camps, and 49% were living in rural areas. Forty one percent of respondents reported living with one or more high-risk person at home, and 53% reported high levels of COVID-19 specific worry (above the mean score of 68).

**Table 1:** Descriptive characteristics of Birzeit University Community COVID-19 study (N = 1851)*.

Characteristic		%

Gender	Men	38
	Women	62
Age (years)	17 to 34	94
	35 to 70	6
District	Center	78
	North	11
	South	7
	Other**	4
Locality	Urban and camps	51
	Rural	49
Status	Undergraduate student	84
	Postgraduate student	10
	Faculty and employee	6
At least one high risk person at home		41
Covid-19 worry scale: Moderate to high (score above 68)		47
Moderate to high levels of distress (score > 50)		40
Moderate to high levels of insecurity (score > 50)		48

* The percentages are weighted, so N was not reported. ** Other: students not permanently living in the West Bank.

**Figure 1:**
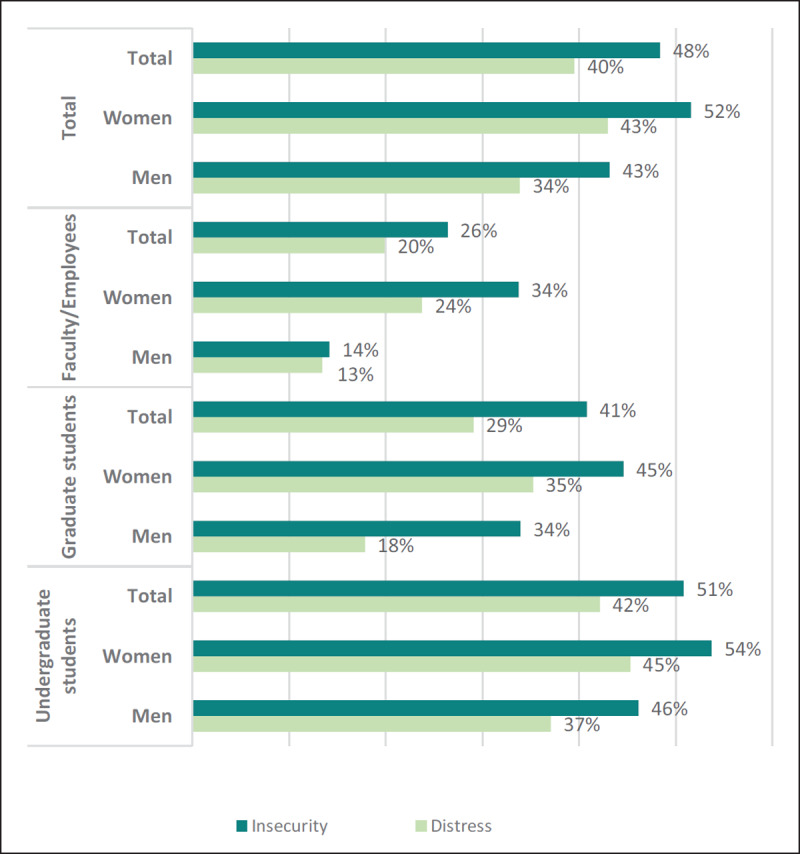
Prevalence of moderate to high levels of distress and insecurity among the Birzeit University community during the COVID-19 lockdown (End March to beginning April 2020) (N = 1,851).

Reported distress and insecurity levels were relatively high. The mean distress score was 47 ± 19 and insecurity score was 50 ± 22. Furthermore, 40% of participants reported moderate to high levels of distress and 48% reported moderate to high levels of insecurity. Figure 1 shows the prevalence of moderate to high distress and insecurity levels by status of participant and gender.

Multiple logistic regression (Table 2) revealed that women had significantly higher odds of reporting distress (OR [95%CI] = 1.63[1.33–1.99]) and insecurity (OR [95%CI] = 1.47[1.2–1.81]) compared to men. Those less than 35 years of age were around two times as likely to report moderate to high levels of distress (OR[95%CI] = 2.31[1.29–4.13]) and almost three times as likely to report moderate to high insecurity (OR[95%CI] = 2.87[1.65–4.99]) compared to those more than 35 years old. Southern West Bankers reported lower levels of insecurity compared to those living in the Center of the West Bank (OR [95%CI] = 0.65[0.45–0.96]). Perceived income was a major predictor of distress and insecurity. Participants who reported good perceived income were 1.27 times as likely to have moderate to high distress (OR[95%CI] = 1.27[1.02–1.58]) and 1.88 times as likely to report moderate to high insecurity (OR[95%CI] = 1.88[1.5–2.36]) compared to those reporting excellent perceived income; while those reporting bad to very bad perceived income were three times as likely to report moderate to high distress (OR[95%CI] = 3.04[2.18–4.25]) and 3.5 times as likely as to report moderate to high insecurity (OR[95%CI] = 3.49[2.45–4.97]) compared to those reporting excellent perceived income.

**Table 2:** Multiple logistic regression models for distress and insecurity among the Birzeit University community during COVID-19 lockdown (End March to beginning April 2020 (N = 1,851)).

		Distress				Insecurity			

		Moderate to high (score > 50)	Unadjusted p-value	Adjusted OR(95% CI)	Adjusted p-value	Moderate to high (score > 50)	Unadjusted p-value	Adjusted OR(95% CI)	Adjusted p-value

Gender	Men	34%	< 0.001	1		43%	< 0.001	1	
	Women	43%		1.63(1.33–1.99)	< 0.001	52%		1.47(1.20–1.81)	< 0.001
Age	17 to 34	41%	< 0.001	2.31(1.29–4.13)	0.005	50%	< 0.001	2.87(1.65–4.99)	< 0.001
	35 to 70	15%		1		21%		1	
District	Center	39%	0.529	1		48%	0.023	1	
	North	42%		1.17(0.87–1.58)	0.304	47%		0.96(0.70–1.31)	0.799
	South	38%		0.90(0.62–1.30)	0.575	44%		0.65(0.45–0.96)	0.028
	Other	45%		1.04(0.65–1.68)	0.866	65%		1.61(0.96–2.72)	0.072
Status	Undergraduate student	42%	< 0.001	1.56(1.13–2.15)	0.006	51%	< 0.001	1.27(0.93–1.75)	0.137
	Faculty/employee/postgraduate student	26%		1		36%		1	
Perceived income	Excellent to very good	31%	< 0.001	1		32%	< 0.001	1	
	Good	39%		1.27(1.02–1.58)	0.034	52%		1.88(1.50–2.36)	< 0.001
	Bad to very bad	62%		3.04(2.18–4.25)	< 0.001	70%		3.49(2.45–4.97)	< 0.001
Covid-19 worry scale	Low (score 0 to 68)	32%	< 0.001	1		31%	< 0.001	1	
	Moderate to high (score 69 to 100)	48%		1.77(1.46–2.14)	< 0.001	68%		4.3(3.53–5.23)	< 0.001
High risk person at home	No	36%	< 0.001	1		46%	0.032	1	
	Yes	44%		1.34(1.11–1.62)	0.003	51%		1.09(0.89–1.33)	0.391

Table 2 also indicates that worry about the pandemic was strongly associated with both distress and insecurity, with those reporting high levels of worry due to the pandemic almost twice as likely to report moderate to high distress (OR [95%CI] = 1.77[1.46–2.14]) and four times as likely to report moderate to high insecurity (OR [95%CI] = 4.3[3.53–5.23]) compared to those reporting lower worry levels. Finally, undergraduate student status and having a high-risk person at home were significantly associated with higher levels of distress but not insecurity. Undergraduate students were 1.5 times as likely to be distressed (OR [95%CI] = 1.56[1.13–2.15]) compared to the other participants, while those living with a high risk person were 1.3 times as likely to report moderate to high distress levels compared to those who do not live with a high risk person.

## Discussion

This exploratory study included a sample drawn from an online survey which included students, employees and faculty members working at a Palestinian University: Birzeit University. The results indicate generally high levels of distress and insecurity among participants. Comparing the results of this study with results on distress obtained for Palestinians living in the Gaza Strip following the 2008–2009 war on the Strip [26], the mean distress in this study was found to be 47 compared to 50 for the Gaza Strip study. Gazans reported more insecurity than West Bankers with a mean of 70 in 2009 compared to 50 in this study [26]. While the Gaza study contained a representative sample of the Strip’s population, our study is restricted to a university community with better living conditions than Gazans. The Gaza Strip has been under a strict siege since 2006, with destruction of property, houses, and land and severe restrictions on travel to the West Bank and abroad. In addition, siege conditions entail the ban on selling goods for export, restrictions on the import of medicines, fuel, basic foods supplies, and building material, with severe ramifications on all aspects of life, compounded by periodic Israeli army attacks and invasions [29]. Nevertheless, the findings for the Birzeit study are significant as they point to generally high levels of distress and insecurity, and indicate the need for intervention.

Women were more likely to report higher levels of insecurity and distress compared to men. This is not surprising as the literature indicates that women tend to report more distress when experiencing difficult life conditions such as becoming an immigrant for example [30], or because, as noted in the literature, women tend to exhibit less distress tolerance than men [31], although this may be the effect of patriarchal and gender relations restricting the agency of women and making them more prone to suffering from distress because of gender constraints on action in order to deal with a problem. In addition, in the Palestinian context, given a gendered division of labor, women are expected to be the primary caretakers of the family, including caring for children, and dealing with their emotional and mental health needs. It is likely that lockdown has increased women’s burdens which may have increased their distress and insecurity levels. A pilot qualitative study in the West Bank indicates that being stuck at home, caring for children, cooking, cleaning, in addition to home schooling are added burdens that women face during lockdown [32], all of which may increase women’s distress and insecurity. Moreover, the literature notes the gendered implication of quarantine. Drawing on experiences in other outbreaks, power differences between men and women have been posited as affecting women’s abilities to access services and have their voices heard [33]. Another study indicates that the closure of schools and nurseries has likely increased pre-existing disparities between men and women in childcare [34]. This raises questions about women being disproportionately affected by lockdown and effects on mental health which should be investigated further.

As we have postulated, participants younger than 35 years were more likely to report higher levels of distress compared to the older generations. While initially, attention was paid to older people because of the higher risk of disease and death with COVID-19, more recent work indicates the need to pay attention to young people and college students. It is reported that the COVID-19 pandemic generates fear and leads to psychosocial problems among college students, including distress due to the uncertainty [14]. Another study suggest that more than 40% of the 14–35 years old youth group studied following the COVID-19 pandemic in China had a tendency to have psychological problems [35]. Our study participants who were 35 years or less and experiencing lockdown had not experienced lockdown previously. In contrast, those more than 35 years old were around 15 years old or more during the Second Palestinian Uprising and have experienced curfews, lockdowns, invasions, and exposure to violence by the Israeli army which they can likely remember clearly [36]. It is possible to suggest that this political curfew experience ameliorates the effects of lockdown on those 35 years old or more who reported less distress and insecurity than the younger participants because they have experienced such situation previously, and know what it means, and how to manage life in such a context. In addition, young adults are still in the process of building their lives and families [37]. The sudden interruption of life, including university education, may well precipitate more distress and insecurity among young adults compared to the older generation because the older generation is already established and has less to lose, while the younger generation is still in the process of finding space in society to function. This entails graduating and finding work, which was already not easy to find before the pandemic given the political context of Israeli occupation and a captive economy; becoming independent of parents, and even beginning to support them in the absence of social protection including social security in old age; getting married, which is expected culturally by the middle twenties, and having children. That is, the sudden interruption of life and studies can mean not being able to establish themselves as full citizens.

The results on Southern West Bankers reporting lower levels of insecurity compared to those living in the Center of the West Bank can be explained in terms of the pandemic reaching the South West Bank later than the center, with the exception of Bethlehem in the South where the disease was first diagnosed [35]. By the time our survey was completed, the Central West Bank was experiencing a rise in cases, while only sporadic cases were spotted in the Southern West Bank, especially in the Hebron District areas. By then, the pandemic was also contained in Bethlehem. However, these results may have been affected by the differences in these regions’ ways of life. The central region of the West Bank is the seat of the PA, where various types of other international and local bodies operate, and is more open to the outside world compared to the other regions [38]. People in the central region may be more exposed to and aware of mental health issues, compared to the Southern Hebron districts, where a more traditional way of life is maintained. Such differences in access, lifestyles, and ways of being could have affected the findings. Clearly, more research needs to be done to explain these results.

The results on income are expected, with those reporting bad or very bad perceived income, and those reporting perceived good income also reporting high levels of distress and insecurity compared to those with excellent perceived income. The gradation in the results of increasing likelihood of distress and insecurity with decreasing perceived income is important to also note. The literature indicates that lockdown during the pandemic can lead to negative mental health effects which, if compounded by poverty, can create major negative mental health consequences [39,40].

Undergraduates reported significantly more distress than graduates or faculty and employees, but not insecurity. These results are understandable as the age of undergraduates tends to be between 17 and 23 years with this generation experiencing lockdown for the first time. This age group can also suffer due to increasing distance between people and absence of communication with their peers, and academic delays [41], and because of the sudden change to online classes which neither faculty nor students have been prepared for[42].

Results indicate that reports of being worried about the COVID-19 pandemic are strongly associated with distress and insecurity. Previous experiences with infectious disease outbreaks, such as the Ebola, or human Avian influenza outbreaks, point to worry and distress as part of people’s reactions to the outbreaks of disease [43,44]. What is important to note is the need to pay attention not only to addressing worries and distress among persons, but also among communities, as this has been shown for neighborhoods [43], and in our case, specific groups in the Birzeit University community who seem to be more negatively affected by the context than other groups. Our experience in mental health interventions also indicates that group work tends to help people realize that they are not alone, and that the problem is not inside the person, but rather the outcome of contextual factors, and may be an expected outcome of the pandemic and lockdown. We have in the past found group work to be effective in situations where entire groups or communities were exposed to political violence. This does not take away the need for work with persons. Rather, this is an alert that one has to be careful about what interventions to use, why, and when.

Respondents who reported having a high risk person at home were more likely to report distress but not insecurity. The same result was found for undergraduate students. It is understandable that people with family members at high risk would report feelings of distress. This has been shown for family caregivers of cancer patients [45], and for entire families where someone in the family is undergoing palliative care [46]. It is not clear why undergraduate students with a high risk person at home reported higher levels of distress. This may be due to age, with young people not used to the potential loss of a loved one. But it could also be due to the dependence of undergraduates on parents for livelihood until they graduate and begin to earn incomes on their own. Further investigation needs to be completed to better understand this result.

At the broader level, this study on the mental health of the Birzeit University community during the COVID-19 pandemic is an example of what happens to people already burdened by military occupation, constraints on the economy, chronic exposure to political violence and lack of freedom, and who are now also experiencing the effects of the pandemic on life, the economy, and health. Given the restrictions imposed on the PA because of the political context, the lack of sovereignty over land, people and resources, the heavy reliance on international aid, and the availability of very limited capacity to respond, lockdown was the only measure it could use to contain the pandemic. This worked well at first, with about 450 infection cases reported from March till end of May among about 2.5 million Palestinians on the West Bank [47]. However, by the end of May, people began to protest lockdown, with poverty rising among an already poor population. According to the World Bank [48], the pandemic presents extraordinary challenges with severe socioeconomic consequences on the Palestinian economy which is already struggling. Job losses, especially in the informal sector which absorbs about 60% of the workforce, is producing a ‘new poor’ category, in addition to those who have already been living in poverty, and where social protection is absent. Thus protests and pressures obliged the PA to ease restriction, and eventually remove all restriction by August of 2020, with the exception of banning wedding and funerals.

From the beginning of June till September 3, 2020, during the second three months of the spread of the virus, the West Bank experienced a serious spike in confirmed cases of COVID-19. Cases jumped from 450 at the end of May to 31,348 cases, with 184 deaths, and with confirmed infections continuing to be on the rise. Added to the problems of poverty and unemployment pushing for the removal of lockdown is the problem of isolation and sanitary measures which are crucial preventive measures, yet difficult to adhere to. This is because those who live in poverty usually live in small, ill equipped and crowded homes and do not have the means to follow sanitary instructions. Moreover, The Palestinian population has always relied on social networks, support, and solidarity for survival in politically turbulent times. Thus social isolation is an unfamiliar notion, and does not make sense to people given their lived experience and needs especially in times of hardship. This can explain not only protests against lockdown because of the necessity of working and bringing income to families given the lack of social protection, but also why people defy the ban on weddings and funerals and continue to hold and attend such gatherings.

## Strengths and limitations

The main strength of this study is shedding light on how the pandemic is affecting mental health as measured by distress and insecurity in a situation where people’s mental health is already compromised because of chronic exposure to political violence. Weaknesses included the fact that online surveys cannot be randomized, with randomization usually used to decrease selection bias, and improve accuracy of the results. This lack of randomization may have affected responses, for example, with fewer employees than their natural distribution at the University filling the survey. The survey excluded guards and cleaners who do not have such electronic accounts and may have reported higher levels of distress and insecurity compared to those who filled the survey. Several possible associated factors were not included as online surveys need to be short and quick to fill. The inclusion of other possible associated factors such as family size, total rooms at home, number of people working in the family, and exposure to political violence during the pandemic, among others, would have strengthened the results.

## Conclusion

We hope that this exploratory study will contribute to understanding how the COVID-19 pandemic and an already challenging context are affecting mental health. It tells the story of a Palestinian community which is caught in double captivity, Israeli military rule of the West Bank compounded by the pandemic; and for Palestinian women, triple captivity, given patriarchal structures of domination and the gendered division of labor. It points to different population groups being affected differently. To date, little attention has been paid to the mental health effects of the pandemic locally among an already compromised Palestinian population. From a public health point of view, it is important to intervene and address these mental health problems, especially that the literature indicates that stress and insecurity are associated with non-communicable diseases and psychiatric disorders [49,50,51]. By exposing distress and insecurity and bringing mental health problems to attention, we hope that this study will inform the policies and interventions of the Palestinian Authority, local non-governmental organization, international groups working in the area, and beyond.

## Additional File

The additional file for this article can be found as follows:

10.5334/aogh.3007.s1 Supplement. Distress and insecurity scale items and scoring.
