# Supplementary material for: Double Burden of COVID-19 Pandemic and Military Occupation: Mental Health Among a Palestinian University Community in the West Bank
Source: Ann Glob Health. 2020 Oct 8;86(1):131. doi: 10.5334/aogh.3007 (PMC7546105; doi:10.5334/aogh.3007)
Supplement: Supplement. — Distress and insecurity scale items and scoring. [file agh-86-1-3007-s1.pdf]

Supplement: distress and insecurity scale items and scoring

Distress scale items (12 items) :

A03.a. To what extent did you feel unable to control the important things in your life?
A03.b. To what extent did you feel unable to cope with all the things that you had to do?
A03.c. To what extent did you feel worried?
A03.d. To what extent did you feel frustrated?
A03.e. To what extent did you feel incapacitated?
A03.f. To what extent did you feel humiliated?
A03.g. To what extent did you feel lonely?
A03.h. To what extent did you feel anxious?
A03.i. To what extent did you feel sad?
A03.j. To what extent did you feel angry?
A03.k. To what extent did you feel fed up with life?
A03.l. To what extent did you feel unable to perform daily activities as usual?

(Scale 0: never, 1: sometimes, 2: most of the time, 3: always, 4: don't know)

Insecurity scale items (10 items):

A01.a. To what extent do you fear for yourself in your daily life?
A01.b. To what extent do you fear for your family in your daily life?
A01.c. To what extent do you feel worry/fear not being able to provide your family with daily life necessities?
A01.d. To what extent do you worry/fear about losing your source of income or your family's source of income?
A01.e. To what extent do you worry/fear losing your home?
A01.f. To what extent do you feel worry/fear from displacement or uprooting?
A01.g. To what extent do you worry/fear for your future and your family's future?

A02.a. To what extent do you feel fear on your safety?
A02.b. To what extent do you feel fear on the safety of your family?
A02.c. To what extent does your family feel fear on your safety?

(Scale 0: never, 1: sometimes, 2: most of the time, 3: always, 4: don't know)

Scoring

a. Insecurity
   1. Code category 4 “do not know” as missing
   2. Sum the 10 variables (A01 (a-g) and A02 (a-c)), you will get a scale between 0 to 30.
   3. To get a scale of 100, divided the new variable by 30 and multiply by 100.
   4. The scale can be used as continuous variable, or can be divided into 2 or more categories , based on the goal of analysis.

b. Distress (A03)
   1. Code category 4 “do not know” as missing
   2. Sum the 12 variables (A03 (a-l)), you will get a scale of 0 to 36.
   3. To get a scale of 100, divided the new variable by 36 and multiply by 100.
   4. The scale can be used as continuous variable, or can be divided into 2 or more categories , based on the goal of analysis.

Notes:

- Some scale items may have the “do not know” response; these should be coded as missing.
- The scale should be calculated based on full responses for all scale questions, (i.e 10 items for human insecurity scale and 12 items for Distress scale).
- However, if there are high number of user- missing or “do not know” responses for selected scale items, the score can be calculated based on valid responses for each participant for valid scale item response. If this to be done, the overall distribution of the scale should be compared to the full responses scale to make sure there is no major deviation, and also characteristics of the missing/do not know scale items should be checked.
